# Rhizosphere soil fertility and microbial community characteristics of *Arundo donax* cv. Lvzhou No.1 in coastal saline-alkali soils

**DOI:** 10.3389/fpls.2026.1745488

**Published:** 2026-02-24

**Authors:** Yuan Luo, Lin Zhang, Yaojin Wang, Weizhen Huang, Yiting Lu, Simeng Song, Jieyi Zhu, Hengyu Zhou, Dewei Su, Dan Zheng, Lin Luo, Hatungimana Mediatrice, Zhanxi Lin, Dongmei Lin

**Affiliations:** 1 College of Life Science, Fujian Agriculture and Forestry University, Fuzhou, China; 2 National Engineering Research Center of Juncao Technology, International College of Juncao Science, Fujian Agriculture and Forestry University, Fuzhou, China; 3 Life Sciences Institute, Zhejiang University, Hangzhou, China; 4 Plant and Microbial Biotechology Program, Rwanda Agriculture and Animal Resources Development Board, Huye, Rwanda

**Keywords:** *Arundo donax*, microbial community, microbial diversity, saline-alkali soils, soil fertility

## Abstract

Saline-alkali soils are widespread in ecologically fragile regions and are characterized by high salinity and alkalinity, leading to soil degradation and reduced productivity. To evaluate the potential of *Arundo donax* cv. Lvzhou No.1 for improving coastal saline-alkali soils, this study was conducted on Pingtan Island, Fujian Province, China. Three treatments were established: a blank control (CK), rhizosphere soils from one-year cultivation (R1), and five-year cultivation (R5). Soil physicochemical properties and microbial community structure were assessed using soil chemical analyses and high-throughput sequencing. Cultivation of *A. donax* cv. Lvzhou No.1 alleviated saline-alkali stress by reducing soil pH and salinity, with stronger effects under long-term cultivation. Soil fertility increased markedly, with organic matter (OM) and total nitrogen (TN) rising by 91.00% and 70.00%, respectively. Microbial diversity also increased, with fungal communities dominated by Ascomycota and bacterial communities by Proteobacteria. Functional predictions showed higher abundances of saprophytic genera (*Acremonium, Fusarium*) and enhanced bacterial metabolic pathways, including fatty acid synthesis and the tricarboxylic acid cycle, indicating increased microbial metabolic activity. These changes promoted organic matter turnover and nutrient release. Canonical correspondence analysis identified OM, TN, available nitrogen (AN), and available phosphorus (AP) as the primary drivers shaping microbial community structure. Overall, long-term cultivation of *A. donax* cv. Lvzhou No.1 improves coastal saline-alkali soils by enhancing physicochemical properties and optimizing microbial community composition. These findings provide a scientific basis for ecological restoration and sustainable utilization of coastal saline-alkali lands.

## 1 Introduction

Driven by factors such as climate change, agricultural over-intensification, and improper soil management, global soil degradation is intensifying, leading to an increasing scarcity of arable land resources and posing threats to both food security and ecological stability (Karlen and Rice, 2015; Zhou and Duan, 2021). Currently, approximately 900 million hectares of land worldwide are affected by salinization, with China accounting for about 78 million hectares (Seydehmet et al., 2018). The rational development and utilization of marginal lands, including saline-alkali soils, can help alleviate pressure on cultivated land, reduce competition with food crops for land and water resources, and safeguard the cultivated land red line (Saifullah et al., 2018). Coastal saline-alkali soil, as a significant land resource with agricultural development potential, exhibits characteristics such as high salinity, high pH, low organic matter content, nutrient deficiency, and low nutrient availability due to long-term periodic immersion by seawater tides (Jiang et al., 2019; Saifullah et al., 2018; Sun et al., 2025). Excessive salt content, particularly exchangeable Na^+^, disrupts soil aggregate structure, resulting in a loose structure with poor stability that diminishes the soil’s capacity to retain water and nutrients, thereby reducing nutrient availability (Amini et al., 2016; Dong et al., 2020). Simultaneously, high osmotic pressure induces physiological water stress in plants, inhibiting their growth and development (Rengasamy, 2010). The accumulation of salt ions leads to soil alkalization, further deteriorating its physicochemical properties and severely limiting the agricultural production potential of coastal saline-alkali soils.

The core of saline-alkali soil reclamation lies in mitigating environmental constraints and reconstructing the microbial community structure to enable the sustainable utilization of these soils (Gu et al., 2024). Current remediation strategies for coastal saline-alkali soils primarily include phytoremediation, physicochemical amendments, and bio-amendments such as biochar, organic acids, and organic fertilizers (Meki et al., 2022). Among these methods, phytoremediation is regarded as the most ecologically sustainable approach. This technique is typically implemented through the cultivation of halophytic or salt-tolerant plants—such as *Sorghum bicolor* var. dochna, *Suaeda salsa*, *Elytrigia elongata*, and *Helianthus tuberosus* L (Steppuhn and Asay, 2005; Wen et al., 2020; Yang et al., 2025)—to regulate soil salt distribution and enhance physicochemical properties, thereby achieving ecological restoration. The key mechanism underlying phytoremediation is the plant-soil-microbe interaction network. Rhizosphere microorganisms promote plant growth and enhance stress tolerance by secreting growth-promoting substances and activating salt-resistance genes (Fan et al., 2025; Paul et al., 2008; Schalk, 2025). In turn, plants selectively shape the structure and function of microbial communities through root exudates (e.g., soluble sugars, amino acids, and secondary metabolites), thereby establishing a positive plant-soil-microbe feedback system. This system continuously reduces soil salinity, improves nutrient availability, and promotes the recovery and stability of ecological functions in saline-alkali soils (Cao et al., 2025; Huang et al., 2015; Rudrappa et al., 2008; Yu et al., 2025).

Although various plant species have been utilized for the phytoremediation of coastal saline-alkali soils, most of these plants offer limited ecological and forage value, and their biomass utilization efficiency remains low. The introduction of multipurpose species, such as *Arundo donax* cv. Lvzhou No.1, presents a promising alternative to address these challenges. This variety exhibits strong salt-alkali tolerance and high biomass production, thereby providing new opportunities for marginal agriculture and the advancement of the bioeconomy (Jia et al., 2025). *Arundo donax* cv. Lvzhou No.1 is a tall perennial herbaceous plant belonging to the Poaceae family, developed by the National Engineering Research Center of Juncao Technology. This variety has a wide range of applications, including edible and medicinal mushroom cultivation, paper production, biomass energy generation, and more. It also shows significant ecological restoration potential in the improvement of saline-alkali soils (Corno et al., 2014; Lei et al., 2020; Lino et al., 2023; Luo et al., 2025). Field trials conducted in regions such as Inner Mongolia, Shandong, and Xinjiang have demonstrated its strong adaptability and effective remediation capacity. This species can enhance the number of soil microorganisms, optimize microbial community structure, improve soil physicochemical properties, reduce salinity, increase soil fertility, and strengthen ecosystem functions (Cocozza et al., 2020).

Rhizosphere soil microorganisms are a vital component of the soil ecosystem, playing essential roles in nutrient cycling, energy flow, and the maintenance of soil structure and ecological balance (Acosta et al., 2011; Bender et al., 2016). The diversity of these microbial communities serves as a key indicator of soil health while simultaneously enhancing ecosystem functioning by promoting plant growth and improving stress resistance (Insam et al, 1996). Rhizosphere microorganisms and plants engage in complex interactions that jointly influence plant physiological processes and drive soil environmental evolution (Lee et al., 2025; Thakar et al., 2025; Trivedi et al., 2020). Li et al. (2025) isolated phosphate-solubilizing *Bacillus* oceanisediminis Q1 and nitrogen-fixing Acinetobacter indicus Q2, both exhibiting strong salt-alkali tolerance, from saline-alkali soil. Through 1:1 co-cultivation, they developed a composite microbial agent, Q3, which was immobilized using biochar as a carrier. This approach significantly improved the physicochemical properties of saline-alkali soils. Building on this foundation, Chadha et al. (2025) demonstrated that the use of halophilic bacteria can effectively enhance plant growth under salt stress, representing a promising strategy for improving both soil remediation and crop productivity in saline-alkali environments. Therefore, this study uses shifts in rhizosphere microbial diversity as a key indicator to evaluate soil health and assess the remediation efficacy of coastal saline-alkali soils.

Previous studies have primarily focused on the short-term effects of plant salt tolerance physiology and rhizosphere microorganism-soil interactions (Feng et al., 2024). However, research on the long-term succession patterns of key functional microbial groups in these systems remains limited (Rath and Rousk, 2015). Specifically, there is a lack of empirical data on how the rhizosphere microbial community structure evolves with cultivation duration and how these changes influence soil salinity and nutrient dynamics. Based on this, the study proposes the following hypotheses: (1) Compared with unvegetated controls, cultivation of *Arundo donax* cv. Lvzhou No. 1 is associated with improved soil physicochemical conditions, including reduced salinity/alkalinity stress and increased nutrient availability, alongside a concomitant restructuring of rhizosphere microbial communities. (2) These soil-microbe shifts exhibit a duration-dependent pattern, with more pronounced changes after long-term cultivation. To test the hypotheses, this study examined the rhizosphere soils of one-year-old and five-year-old *A. donax* cv. Lvzhou No.1 cultivation sites in coastal saline-alkali soils, with adjacent unvegetated sandy soils as controls. High-throughput sequencing was used to analyze microbial community composition and structural changes under different cultivation durations, focusing on their roles in soil nutrient dynamics and saline-alkali remediation. Additionally, correlation analysis was performed to explore the relationship between soil physicochemical properties and microbial community structure. Overall, this study aims to clarify duration-dependent plant-microbe-soil interactions under *A. donax* cv. Lvzhou No.1 cultivation and to provide scientific evidence supporting its ecological application in coastal saline-alkali regions.

## 2 Materials and methods

### 2.1 Study site description

The experiment was conducted from 2019 to 2024 at the Coastal Juncao Windbreak and Sand-Fixation Base, situated west of the Aoqian Roll-on/Roll-off Terminal in Pingtan, Fujian Province, China (25°28’N, 119°50’E). The study area has a subtropical maritime monsoon climate, characterized by a mean annual temperature of 19.60 °C and distinct dry and rainy seasons. The rainy season lasts from March to June, with an average annual precipitation of 1172 mm, which is lower than the mean annual evaporation of 1300 mm, thereby classifying the region as a low-precipitation zone within Fujian Province. The terrain is high in the north and south but lower in the middle, with a maximum elevation of 434.60 m. The coastline is irregular and diverse, consisting mainly of bedrock erosional, lateritic erosional, and sandy lagoonal depositional coasts. Vegetation in the area is dominated by anthropogenic plant communities undergoing retrogressive succession, resulting in low species diversity and a simple community structure. Typical coastal species include *Eurya emarginata*, *Oenothera drummondii*, and *Atriplex maximowicziana*. Due to seawater backflow during high tides, the region has experienced prolonged marine erosion, leading to the formation of coastal salinized fluvo-aquic soil.

### 2.2 Plant materials

The experimental plant was *Arundo donax* cv. Lvzhou No.1, provided by the China National Engineering Research Center of Juncao Technology. Healthy seed stems with plump buds were selected, soaked in water for 24–36 h to promote sprouting, and planted at a 45° angle with a spacing of 80 cm × 80 cm.

### 2.3 Experimental design and sample collection

Field plots planted with *A. donax* cv. Lvzhou No.1 for one year (R1; established in 2024) and five years (R5; established in 2019) were selected as the experimental sites. Enclosed bare sandy land devoid of *A. donax* cv. Lvzhou No.1 served as the control (CK). Each treatment consisted of five independent plots, each measuring 20 m × 20 m. Prior to the establishment of the plots, all sites (CK, R1, and R5) were situated on unmanaged wasteland with comparable initial land conditions. To minimize fertilization as a confounding factor when assessing planting-duration effects, fertilizer inputs (type, timing, and total rates) were standardized across treatments. All plots received organic fertilizer (15,000 kg ha^-^¹) as a basal dressing in the establishment year (2019 for R5 and CK; 2024 for R1). A single topdressing of urea (45 kg ha^-^¹) and diammonium phosphate (30 kg ha^-^¹) was applied at the tillering stage of planted stands; CK plots received the same fertilizers and rates within the same calendar-date window. All fertilizers were evenly applied using identical procedures, and no further fertilization was conducted thereafter.

Soil sampling was conducted in June 2024 using a five-point sampling method. Five sampling points were selected within each plot, corresponding to five individual *A. donax* cv. Lvzhou No.1 plants in the R1 and R5 treatments, while equidistant points were used in the CK plots. Following the method described by Li et al. (2023), surface plant residues were first removed during sampling. Plant roots were then carefully excavated, gently shaken and tapped to remove loosely adhering soil, and the soil tightly attached to the root surface was collected as rhizosphere soil. Residual soil on the roots was further brushed off using a sterile brush. The collected soil samples were placed into sterile sealed bags and immediately stored in an ice box.Upon returning to the laboratory, soil samples from the five sampling points within each plot were thoroughly homogenized and combined into one composite sample, resulting in five composite samples for each treatment. A portion of each sample was stored at −80 °C for microbial community analysis, while the remaining samples were air-dried at room temperature for the determination of soil physicochemical properties.

### 2.4 Analysis of soil chemical properties

Organic matter (OM) was quantified using the potassium dichromate method, while total nitrogen (TN) was assessed through the Kjeldahl method (Wang et al., 2024b). Total phosphorus (TP) and available phosphorus (AP) were analyzed via the molybdenum-antimony colorimetric method and spectrophotometry, respectively (Kowalenko and Babuin, 2007; Wang et al., 2024b). Available nitrogen (AN) was measured using the alkaline diffusion method (Cheng et al., 2023). Total potassium (TK) and available potassium (AK) were determined through flame photometry, with TK being measured after digestion with a mixed acid solution (H_2_SO_4_;-HCl) and AK extracted using 1 M ammonium acetate (NH_4_;OAc, pH 7.0) prior to measurement (Shao et al., 2020). Soil pH and electrical conductivity (EC) were measured at soil-to-water (mass: volume) ratios of 1:2.5 and 1:5, respectively.

### 2.5 High-throughput sequencing and biological analysis of soil microbiota

Soil microbial DNA was extracted using the E.Z.N.A.^®^ Soil DNA Kit (Omega Bio-tek, USA) according to the manufacturer’s instructions. The concentration and purity of DNA were determined using a NanoDrop 2000 UV-Vis spectrophotometer (Thermo Scientific, USA), and DNA quality was assessed by 1% agarose gel electrophoresis. The bacterial 16S rRNA gene was amplified using primers 338F (5′-ACTCCTACGGGAGGCAGCA-3′) and 806R (5′-GGACTACHVGGGTWTCTAAT-3′), while the fungal ITS region was amplified with primers ITS1F (5′-CTTGGTCATTTAGAGGAAGTAA-3′) and ITS2R (5′-GCTGCGTTCTTCATCGATGC-3′). After PCR amplification, the products were purified by agarose gel electrophoresis, and sequencing libraries were constructed using the TruSeq Nano DNA LT Library Prep Kit (Illumina, USA). High-throughput sequencing was subsequently performed on the Illumina platform (Shanghai Paiseeno Biotechnology Co., Ltd.).

The sequencing data were processed using the QIIME 2 (version 2019.4) platform. After removing primers from the raw sequences using cutadapt, quality control, denoising, merging, and chimera removal were performed using DADA 2, generating amplicon sequence variants (ASVs). After merging the ASVs from all libraries, singleton ASVs with a total abundance of 1 across all samples were removed.

Taxonomic annotation of bacterial ASVs was based on the SILVA database (version 138.1), and taxonomic annotation of fungal ASVs was based on the UNITE database (version 9). Both annotations were performed using the classify-sklearn Naive Bayes classifier from the feature-classifier plugin in QIIME 2, with a classification confidence threshold set at 0.7. The filtered ASV abundance matrix was utilized for subsequent community structure analysis, diversity analysis, and statistical tests.

### 2.6 Data processing and statistical analysis

Alpha diversity analysis was performed using R software and QIIME 2, and Beta diversity was assessed using UniFrac distance metrics. Functional prediction of ASV data was carried out using PICRUSt 2, and microbial diversity analysis was performed on the Paiseeno cloud platform. Differences in microbial community structure among treatments were evaluated using permutational multivariate analysis of variance (PERMANOVA) based on Bray-Curtis dissimilarity, implemented with the adonis 2 function in the vegan package in R (v4.5.0). Canonical correspondence analysis (CCA) was performed in R to examine the relationships between soil microbial communities and soil physicochemical properties. Microbial co-occurrence networks were constructed in R based on Pearson correlation analysis, with a correlation coefficient threshold of 0.6 and a significance level of *p* < 0.001. The network visualization was achieved using Cytoscape (v3.10.0). All raw data were statistically analyzed using Excel 2010. One-way analysis of variance (ANOVA) and Duncan’s multiple comparison test were performed using SPSS 22.0, with statistical significance determined at *p* < 0.05. Graphical representations were generated using Origin 2021 and Canoco 5.

### 2.7 Sequence accession numbers

The raw high-throughput sequencing data have been submitted to the NCBI database with BioSample numbers SAMN54407995 (CK), SAMN54407996 (A, R1), and SAMN54407997 (B R5) with BioProject number PRJNA1396986.

## 3 Results and analysis

### 3.1 Effects of different growing years of *Arundo donax* cv.Lvzhou No.1 on soil nutrients

The physicochemical properties of the soil are summarized in **Table 1**. Compared to the CK the cultivation of *A. donax* cv. Lvzhou No.1 resulted in a significant decrease in soil pH, with the reduction becoming increasingly pronounced over the duration of planting. Although the EC value initially decreased before rising again, it consistently remained lower than that of the CK group. In terms of nutrient content, the levels of OM, TN, and AN saw significant increases, with values approximately 47.67%, 41.18%, and 44.30% higher than those in the CK group, respectively. The R5 group exhibited significantly higher TP and TK contents compared to the R1 group, with increases of approximately 9.10% and 19.63%, respectively; however, no significant differences were observed between R5 and CK. The AP content was highest in the R1 group, showing significant differences from both CK and R5, while the AK content was most prominent in R5, demonstrating significant differences compared to CK.

**Table 1:** Chemical properties of soil for Arundo donax cv.Lv zhou No.1 in coastal saline-alkali land at different planting years.

Measures	pH	Ec (mS/cm)	OM (g/kg)	TN (g/kg)	TP (g/kg)	TK (g/kg)	AP (mg/kg)	AK (mg/kg)	AN (mg/kg)
CK	9.21 ± 0.07 a	86.82 ± 9.24 a	1.57 ± 0.13 c	0.10 ± 0.00 c	0.34 ± 0.01 ab	2.29 ± 0.20 ab	5.28 ± 0.48 b	47.78 ± 4.70 b	9.23 ± 1.49 c
R1	9.11 ± 0.05 b	73.28 ± 2.97 b	2.50 ± 0.33 b	0.14 ± 0.01 b	0.33 ± 0.01 b	2.14 ± 0.14 b	6.12 ± 0.42 a	52.87 ± 0.10 ab	12.35 ± 2.41 b
R5	9.07 ± 0.04 b	83.2 ± 5.93 a	3.00 ± 0.21 a	0.17 ± 0.00 a	0.36 ± 0.02 a	2.56 ± 0.30 a	5.07 ± 0.34 b	66.62 ± 16.73 a	16.57 ± 2.44 a

Values are means ± SD (n = 5 replications). CK, the blank control; R1, one-year cultivation; R5, five-year cultivation. Different lowercase letters in the same column indicate significant difference used by Duncan’s new multiple range test (*p* < 0.05).

### 3.2 Analysis of soil microbial sequences and community differences

The dilution curves of microorganisms in saline-alkali soils, observed over varying cultivation durations of *A. donax* cv. Lvzhou No.1, along with the control sandy soil, are illustrated in **Supplementary Figure S1**. As sequencing depth increased, the dilution curves for all samples plateaued, indicating that the sequencing was sufficiently deep to accurately capture the microbial community composition. As detailed in **Supplementary Table S1**, significant differences were observed in the number of fungal and bacterial ASVs among the treatments. The CK group exhibited the lowest counts of ASVs, recording 83.98 for fungi and 2,528.30 for bacteria. In contrast, the R1 group demonstrated significant increases, with counts of 184.26 for fungi and 4,841.82 for bacteria. Notably, the R5 group recorded the highest counts, with 233.40 for fungi and 5,187.02 for bacteria.

**Figure 1:**
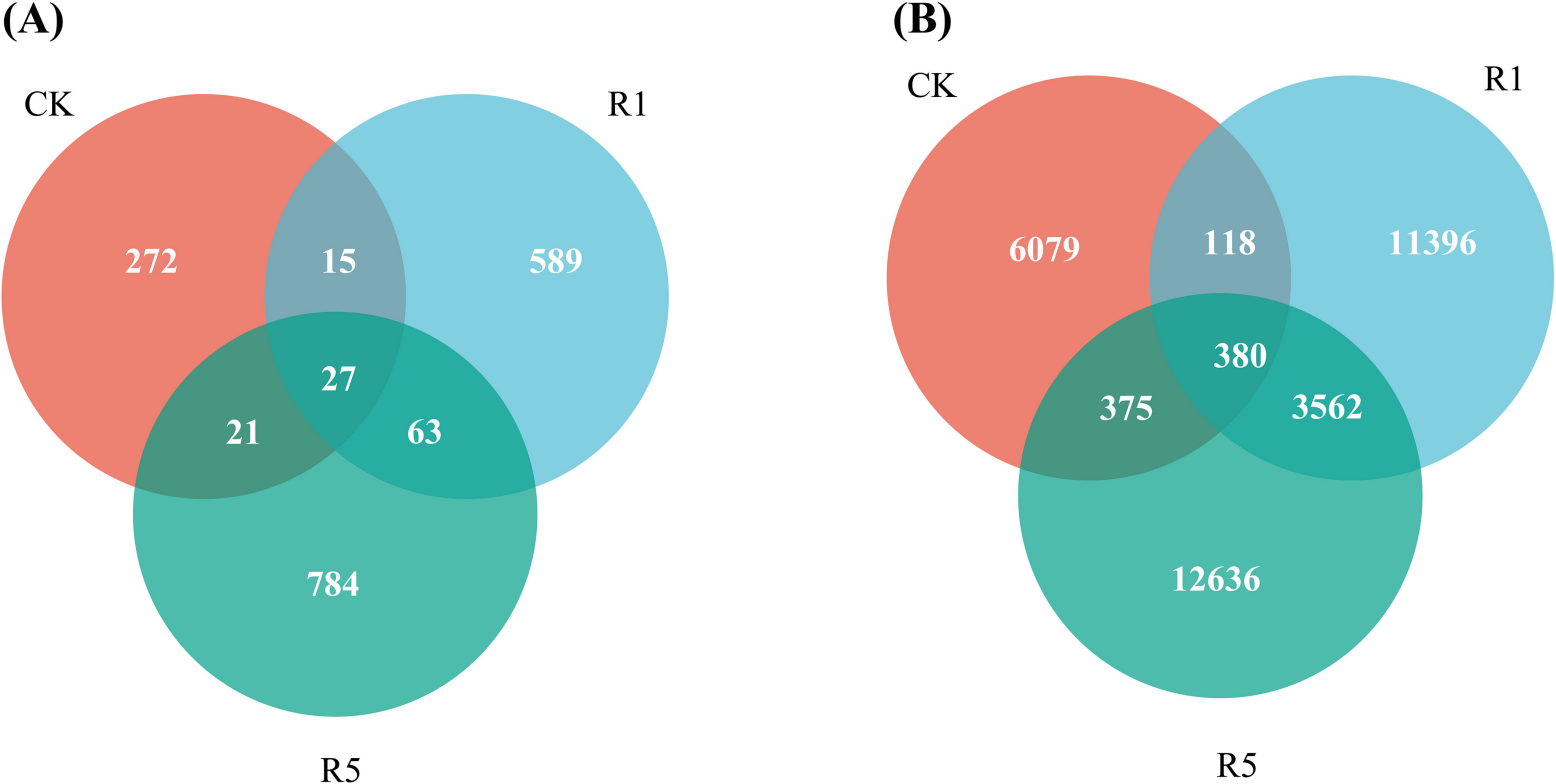
Venn diagram of soil microorganisms in *Arundo donax* cv. Lv zhou No.1 Plots and bare land in saline-alkali land at different planting years. **(A)** Venn Diagram of Fungi; **(B)** Venn Diagram of Bacteria. CK, the blank control; R1, one-year cultivation; R5, five-year cultivation.

Venn diagram analysis at the species level (**Figure 1**) revealed the detection of 1,771 fungal ASVs and 34,546 bacterial ASVs across the three groups. For fungi (**Figure 1A**), the number of unique ASVs was highest in R5 (44.27%), followed by R1 and CK, which shared only 1.52% of the total ASVs. In the case of bacteria (**Figure 1B**), R5 also exhibited the highest number of unique ASVs (36.58%), with 1.10% of ASVs shared across all treatments.

### 3.3 Soil microbial diversity analysis

**Figure 2:**
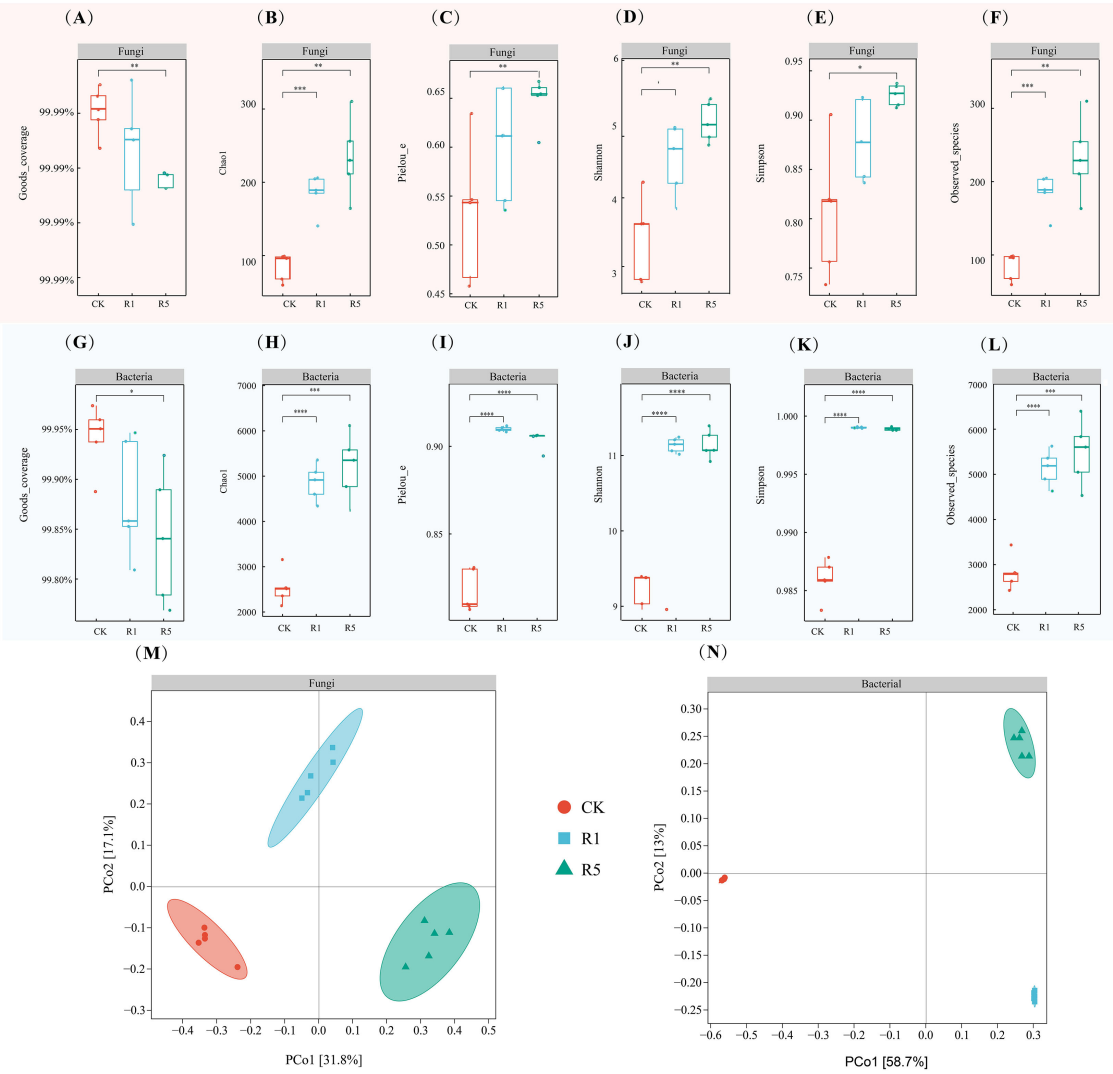
Box plot of alpha diversity of rhizosphere microorganisms in coastal saline-alkali soil planted with *Arundo donax* cv. Lvzhou No.1 for different years. **(A–F)** represent fungi, **(G–L)** represent bacteria. *, **, *** and **** exhibit significance at the levels of *p* < 0.05, *p* < 0.01, *p* < 0.001 and *p* < 0.0001, respectively; Principal coordinate analysis based on the Bray-Curtis distance of soil fungal **(M)** and bacterial **(N)** beta diversity. Values are means ± SD (n = 5 replications). CK refers to the blank control; R1 indicates one years of cultivation; and R5 denotes five years of cultivation.

The microbial diversity indices of soils under different growing years of *A. donax* cv. Lvzhou No.1, along with those of the blank sandy soil control, are presented in **Figure 2**. All samples exhibited Good’s coverage values exceeding 99.80% (**Figures 2A, G**), indicating adequate sequencing depth. Compared to the CK, both R1 and R5 demonstrated higher microbial richness and diversity (**Figures 2B–F, H–L**). The Chao1 index (**Figure 2B**) revealed that R5 enhanced fungal and bacterial richness relative to CK by approximately 64% and 51%, respectively. Similarly, Pielou evenness (**Figure 2K**) increased under R5 by around 18% (fungi) and 10% (bacteria), while Shannon diversity (**Figure 2L**) rose by approximately 33% and 17%. Throughout the cultivation durations, diversity indices in R5 were generally higher than those in R1 (with the exception of bacterial Pielou evenness), although these differences were not statistically significant. Collectively, cultivation was associated with increased rhizosphere microbial richness and diversity, with more substantial numerical increases observed with longer planting durations.

At the ASV level, Principal Coordinates Analysis (PCoA) based on Bray-Curtis distances was employed to assess β-diversity (**Figures 2M, N**). For fungi, PC1 and PC2 accounted for 31.80% and 17.10% of the variation, respectively (**Figure 2M**); for bacteria, PC1 and PC2 explained 58.70% and 13.00% (**Figure 2N**). Samples from CK, R1, and R5 formed distinct clusters in both ordinations, indicating compositional differences among treatments and planting durations. Consistently, Permutational Multivariate Analysis of Variance (PERMANOVA; **Supplementary Table S2**) demonstrated significant treatment effects on community structure, with cultivation accounting for 47.69% of the variation in fungal communities (R² = 0.48, *p* = 0.001) and 71.63% in bacterial communities (R² = 0.72, *p* = 0.001). This indicates that the cultivation of *A. donax* cv. Lvzhou No.1, as well as its different planting durations, significantly influenced the composition of the soil microbial community.

### 3.4 Analysis of rhizosphere soil microbial community composition

**Figure 3:**
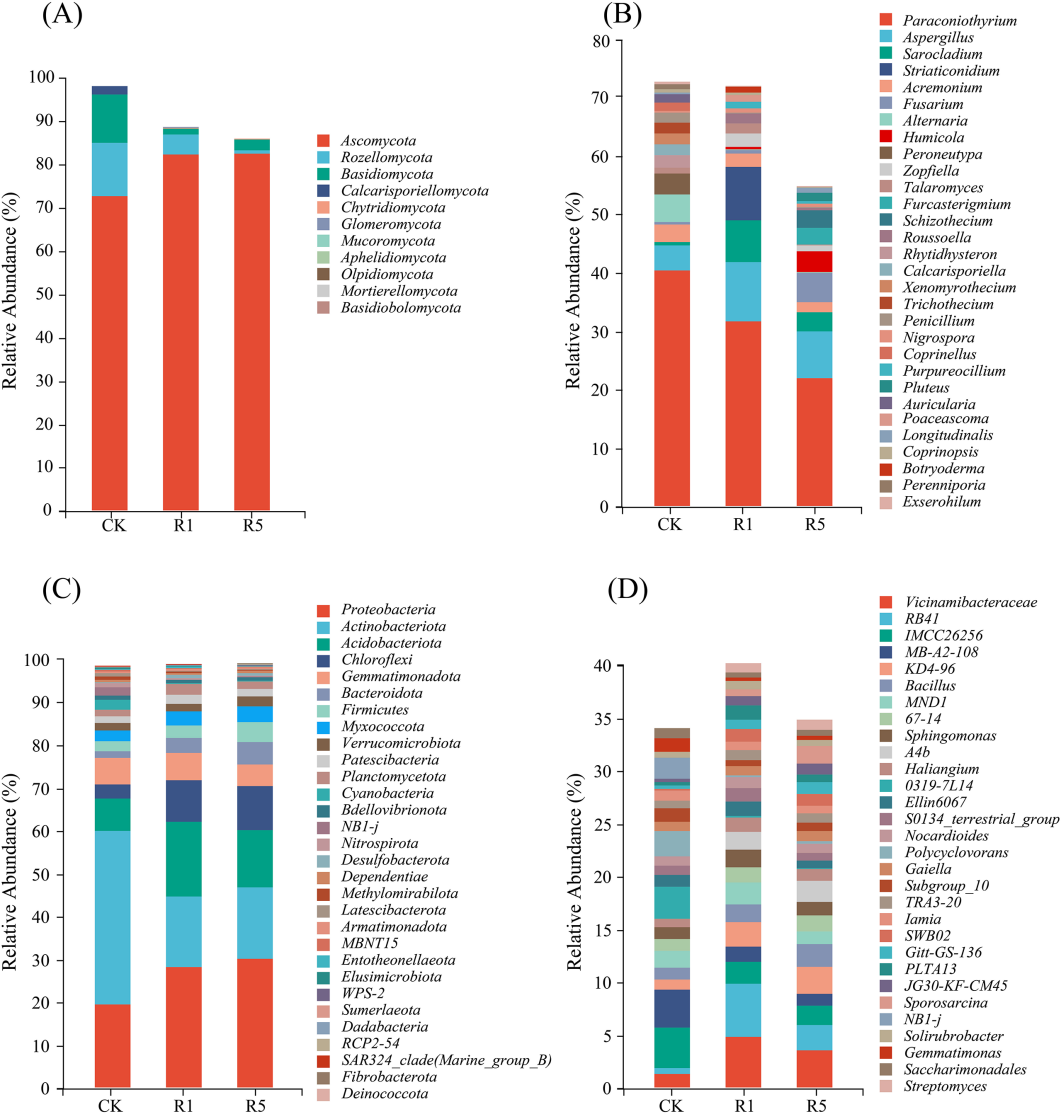
The relative abundances of the top30 of major taxa at the levels of fungal **(A)** and bacterial **(C)** phyla, as well as fungal **(B)** and bacterial **(D)** genera, are presented for coastal saline-alkali land planted with *Arundo donax* cv. Lvzhou No.1 over different years. CK refers to the blank control; R1 indicates one years of cultivation; and R5 denotes five years of cultivation.

Annotation of valid ASVs identified 13 fungal phyla, 35 classes, 236 genera, and 311 species, as well as 47 bacterial phyla, 146 classes, 1,241 genera, and 3,235 species. Relative abundance plots for the top 30 fungal and bacterial taxa at both the phylum and genus levels were generated (**Figure 3**), with the three most abundant phyla and genera defined as dominant.

Phylum level (**Figures 3A, C**): Across CK, R1, and R5, fungi were mainly represented by Ascomycota, Rozellomycota, and Basidiomycota, with Ascomycota as the most abundant phylum. Compared with CK, the relative abundance of Ascomycota in R1 and R5 increased by approximately 13.81%, whereas Rozellomycota and Basidiomycota decreased significantly by approximately 63.18% and 87.51%, respectively. Bacterial communities were primarily composed of Proteobacteria, Actinobacteriota, and Acidobacteriota. In CK, Actinobacteriota was the dominant phylum (40.84%), but after cultivation of *A. donax* cv. Lvzhou No.1, Proteobacteria became dominant, accounting for over 28%. Concurrently, the relative abundance of Actinobacteriota decreased by approximately 58.18%, while Proteobacteria and Acidobacteriota increased by about 50.52% and 51.39%, respectively.

Genus level (**Figures 3B, D**): The predominant fungal genera identified in this study were *Paraconiothyrium*, *Aspergillus*, and *Sarocladium*. Among these, *Paraconiothyrium* exhibited the highest average relative abundance of 31.18% across all samples. Notably, its relative abundance significantly decreased in samples R1 and R5 compared to the control (CK), while *Aspergillus* showed an increase. Conversely, the abundance of *Sarocladium* was greater in R1 than in both CK and R5. In terms of bacterial genera, the dominant taxa included *IMCC26256*, *MB-A2-108*, and *Vicinamibacteraceae*. *IMCC26256* was most prevalent in CK with a relative abundance of 3.81%, whereas *Vicinamibacteraceae* reached its peak in R1 and R5. From CK to R1 and R5, both IMCC26256 and MB-A2–108 experienced a reduction of approximately 56.32%, while *Vicinamibacteraceae* and the genus RB41 increased by about 399.27%. With the prolonged cultivation of *A. donax* cv. Lvzhou No.1, the relative abundances of *Vicinamibacteraceae* and RB41 declined by approximately 39.69%, whereas *Bacillus* increased by approximately 31.29%.

**Figure 4:**
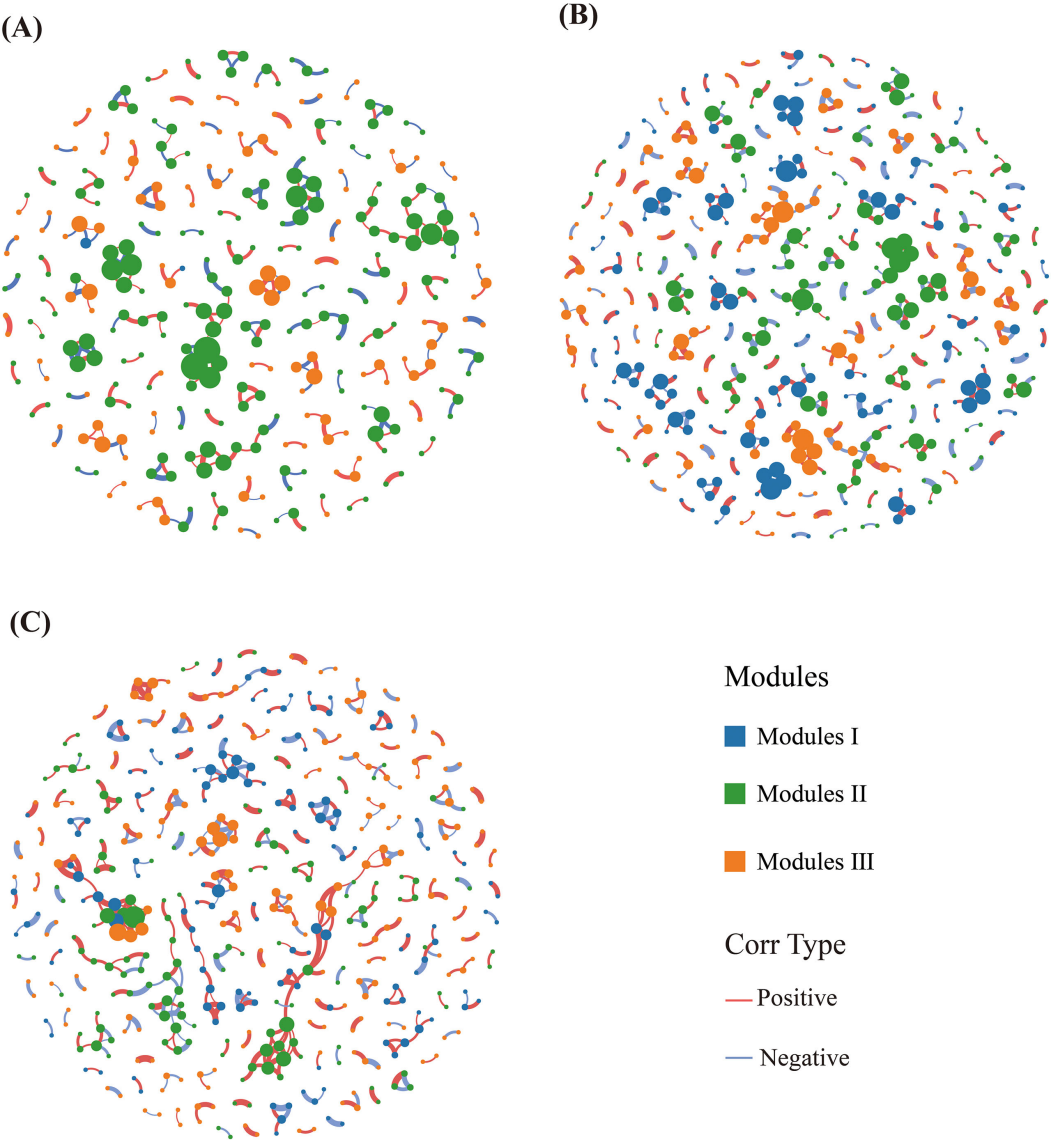
Co-occurrence network analysis of soil bacteria community. Nodes indicate ASVs, and edges (the line between two nodes) indicate the presence of a significant correlation between two ASVs (Spearman *ρ* > 0.6, *p* < 0.001). The same module is represented by the same colour. Mean values of diversity indices were used to calculate the variance across treatments. **(A)** the blank control; **(B)** one-year cultivation; **(C)** five-year cultivation. CK refers to the blank control; R1 indicates one years of cultivation; and R5 denotes five years of cultivation.

Due to the high sparsity and low overlap of fungal ASVs among samples, fungal co-occurrence network analysis was not conducted; instead, only bacterial co-occurrence network analysis was performed (**Figure 4**). With increasing planting duration, both node and edge numbers increased, indicating higher network complexity. Specifically, the CK group had 317 nodes and 248 edges (142 pos. corr. and 106 neg. corr.; **Figure 4A**). The network exhibited low connectivity (average degree of 1.56) and a large path length (network diameter = 7.95). The modules were loose, with low connectivity, reflecting a loosely organized microbial community structure. The R1 group had 493 nodes and 377 edges (220 pos. corr. and 157 neg. corr.), with an average degree of 1.53 and a network diameter of 6.96, indicating relatively higher networkconnectivity (**Figure 4B**). The R5 group had the most 484 nodes and 426 edges (284 pos. corr. and 142 neg. corr.), with an average degree of 1.76 and a network diameter of 9.94 (**Figure 4C**). This network exhibited numerous tightly connected modules and strong overall connectivity, indicating a compact structure and complex network topology. Overall, the cultivation of *A. donax* cv. Lvzhou No.1 significantly influenced the organizational structure of the microbial community, particularly in the R5 group, where the network structure was the most complex.

### 3.5 Analysis of indicator species in microbial communities

**Figure 5:**
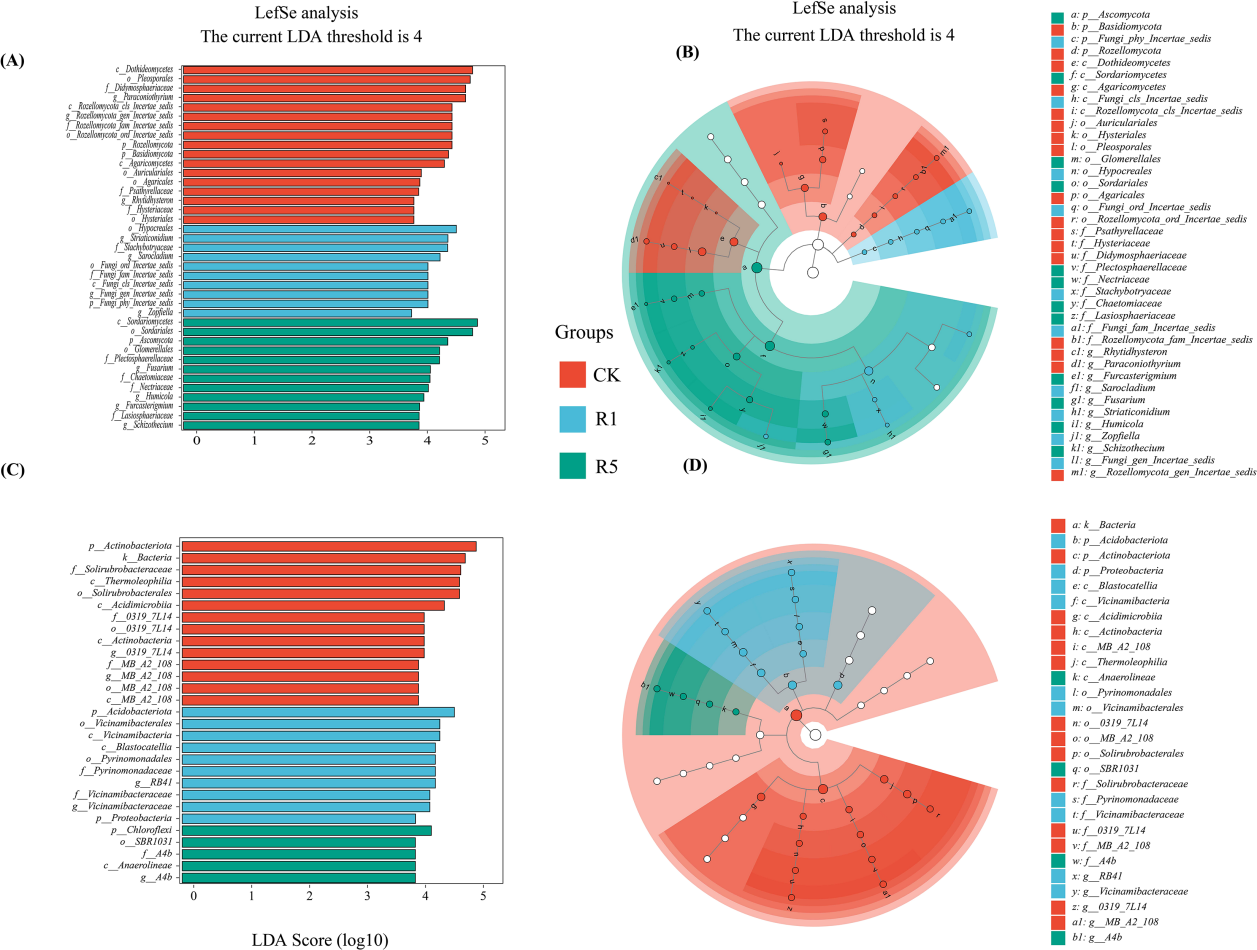
LEfSe analysis of microbial abundance between Rhizosphere microorganisms of *Arundo donax*. cv. Lvzhou No.1 and control group. The LDA score identified the size of fungal **(A)** and bacterial **(C)** differentiation between plateau and control groups with a threshold value of 4. The cladogram of fungil **(B)** and **(D)** microbial communities. CK refers to the blank control; R1 indicates one years of cultivation; and R5 denotes five years of cultivation.

LEfSe analysis was conducted on soil microorganisms from saline-alkali soils under different cultivation durations of *A. donax* cv. Lvzhou No.1 and the unplanted control (CK) to identify significantly differential indicator taxa, using an LDA score > 4 and a relative abundance threshold of 0.01, with taxonomic annotation performed at multiple levels (**Figure 5**).

Fungi (**Figures 5A, C**): A total of 4 phyla, 5 classes, 9 orders, 10 families, and 11 differential genera were enriched. In CK, enriched taxa were mainly concentrated within the phylum Rozellomycota and the genus *Paraconiothyrium*. In R1, significantly enriched taxa included the phylum Ascomycota, the genera *Acremonium*, *Sarocladium*, *Striaticonidium*, and several unclassified taxa. In R5, enriched taxa primarily involved the class Sordariomycetes and the genera *Fusarium* and *Humicola*. Overall, the core differential fungal taxa in CK were clearly distinct from those in R1 and R5, and the dominant taxa in R1 and R5 exhibited systematic shifts corresponding to cultivation duration.

Bacteria (**Figures 5B, D**): A total of 3 phyla, 7 classes, 6 orders, 6 families, and 5 differential genera were enriched. CK was enriched in taxa associated with the phylum Actinobacteriota, including the class Actinobacteria, the order Solirubrobacterales, and the family Solirubrobacteraceae, as well as the class Thermoleophilia. Additionally, the class Acidimicrobiia and several unclassified taxa (e.g., order 0319-7L14, genus *MB-A2-108*, and taxa associated with *IMCC26256*) showed high abundance in CK. In R1, enriched taxa included the phyla Acidobacteriota and Proteobacteria, the order Pyrinomonadales, and taxa related to Vicinamibacteriota. In R5, dominant taxa comprised the phylum Chloroflexi, class Anaerolineae, and unclassified groups *67-14*, *A4b*, and *KD4-96*. In summary, both fungal and bacterial communities showed clear differentiation among groups, and fungal indicator taxa varied with cultivation duration. In summary, both fungal and bacterial communities showed clear differentiation among groups, and fungal indicator taxa varied with cultivation duration of *A. donax* cv. Lvzhou No.1.

### 3.6 Analysis of microbial community functions

**Figure 6:**
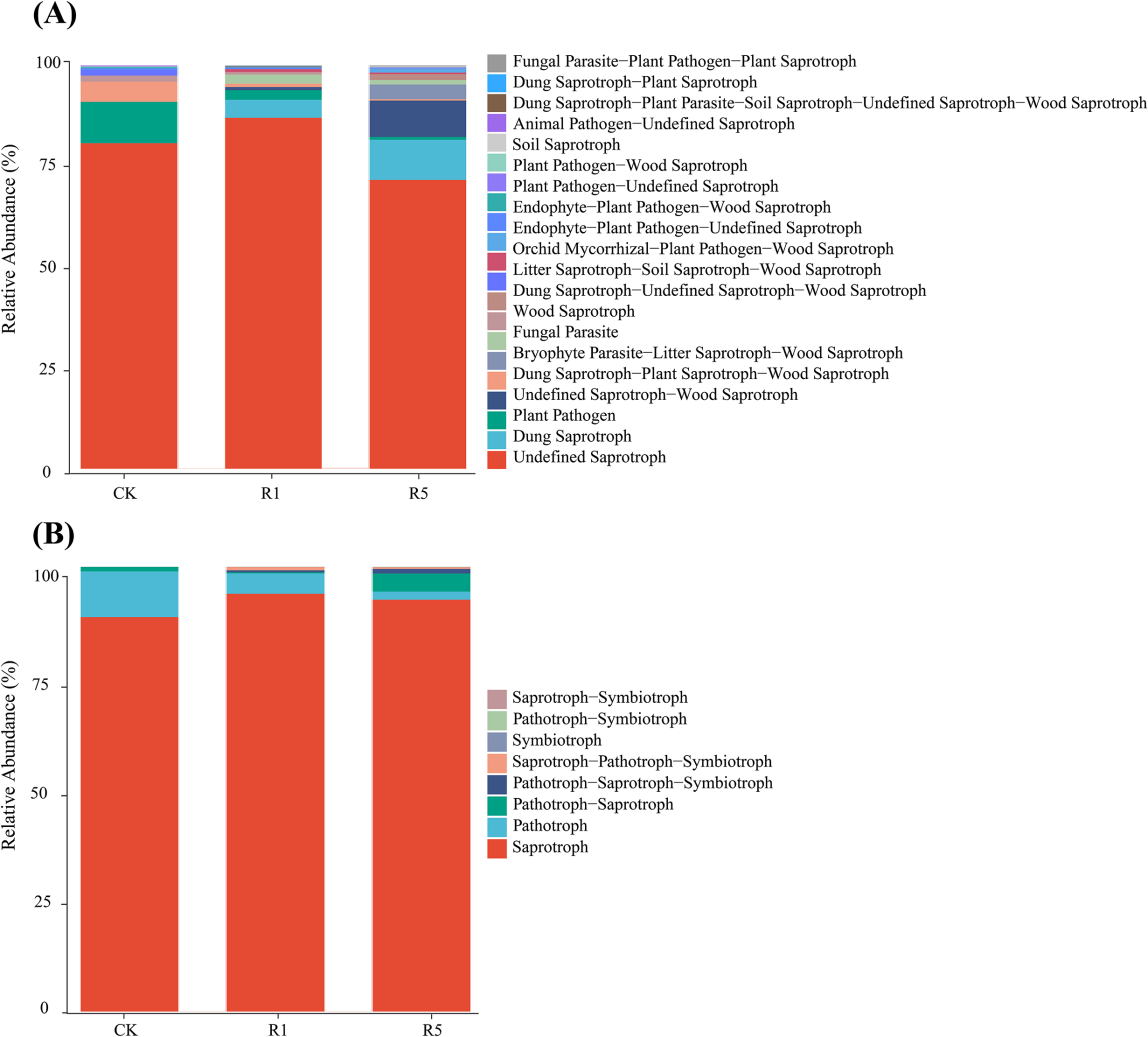
FUNGuild functional classification of soil fungi in *Arundo donax* cv.Lvzhou No.1. **(A)** Relative abundance of fungal guilds; **(B)** Relative abundance of fungal trophic modes. CK refers to the blank control; R1 indicates one years of cultivation; and R5 denotes five years of cultivation.

Fungal community functions were analyzed using the FUNGuild database (**Figure 6**). Functional group composition is shown in **Figure 6A**. In CK, the fungal community was dominated by undefined saprotrophs, plant pathogens, the combined group ‘Dung-Plant Saprotroph-Wood Saprotroph,’ and wood saprotrophs. In R1, the main groups included undefined saprotrophs, dung saprotrophs, plant pathogens, and fungal parasites. In R5, the dominant groups were undefined saprotrophs, dung saprotrophs, the combined group ‘Undefined Saprotroph-Wood Saprotroph,’ and a mixed functional group ‘Bryophyte Parasite-Litter Saprotroph-Wood Saprotroph.’ Compared with CK, the relative abundance of dung saprotrophs gradually increased in R1 and R5, whereas plant pathogens and the ‘Dung-Plant Saprotroph-Wood Saprotroph’ group decreased progressively. Additionally, fungal parasites were less abundant in R5 than in R1, while the relative abundance of the ‘Undefined Saprotroph-Wood Saprotroph’ group increased.

Trophic mode distribution (**Figure 6B**): In both CK and R5, the predominant rhizosphere fungi were classified as Saprotrophs, Pathotrophs, and Pathotroph-Saprotrophs. In R1, the dominant trophic modes included Saprotrophs, Pathotrophs, and the transitional mode Pathotroph-Saprotroph-Symbiotroph. Overall, cultivation of *A. donax* cv. Lvzhou NO.1 increased the relative abundance of Saprotrophs, while the abundance of Pathotrophs decreased. With prolonged cultivation, the abundance of Pathotrophs continued to decline, whereas the relative abundance of the Pathotroph-Saprotroph-Symbiotroph transitional mode increased.

**Figure 7:**
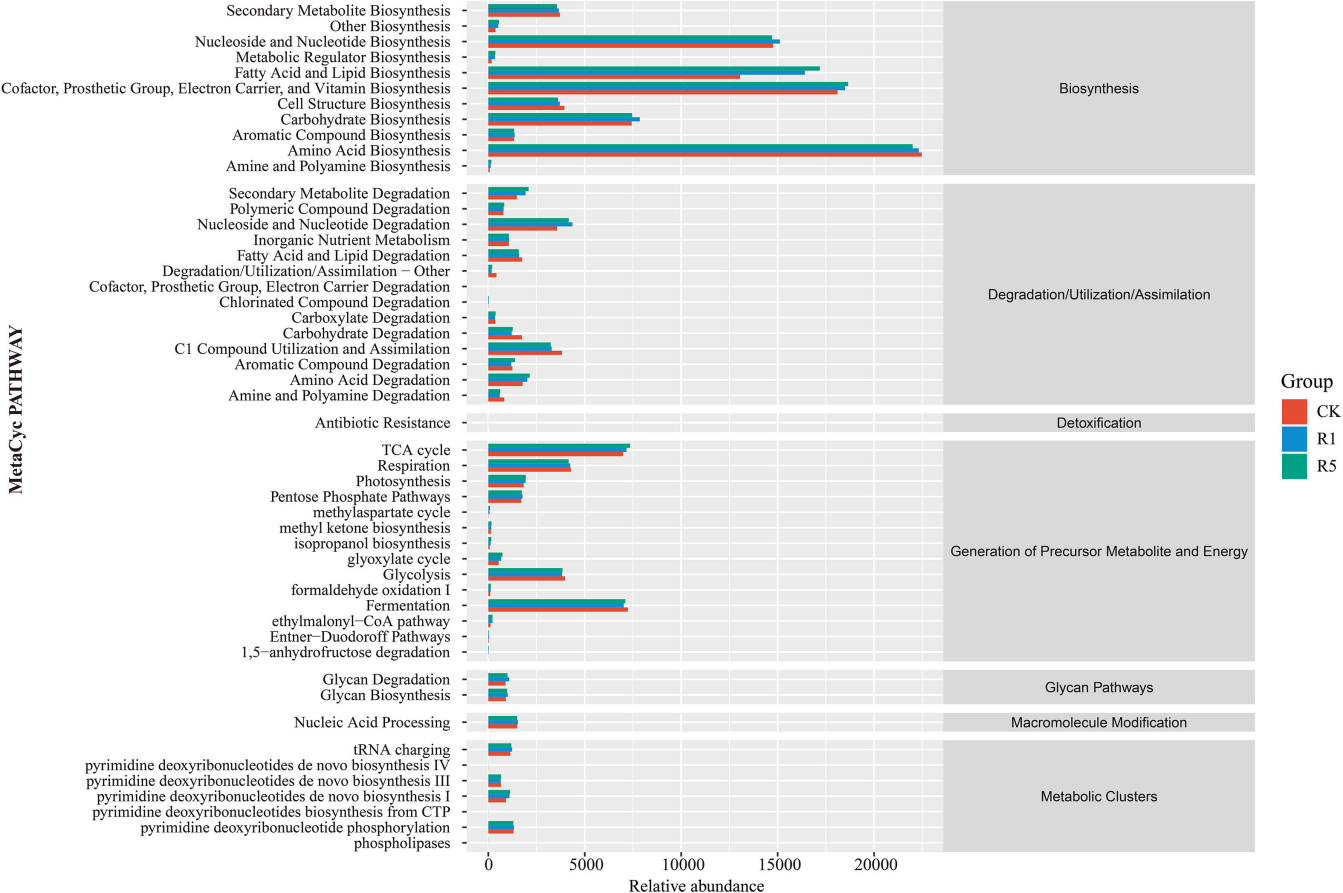
Soil microbial function and ecotype prediction. The rightmost is the first level classification to which the pathway belongs. CK refers to the blank control; R1 indicates one years of cultivation; and R5 denotes five years of cultivation.

Functional annotation of soil bacterial genes was performed using PICRUSt and the KEGG database (**Figure 7**). At the primary functional level, bacterial genes across all samples were primarily categorized into seven functional pathways: Biosynthesis, Degradation/Utilization/Assimilation, Detoxification, Generation of Precursor Metabolites and Energy, Glycan Pathways, Macromolecule Modification, and Metabolic Clusters. At the secondary functional level, the ten most abundant pathways in rhizosphere bacterial communities, ranked by relative gene function abundance, included: Amino Acid Biosynthesis (20.64%–21.08%), Cofactor, Prosthetic Group, Electron Carrier, and Vitamin Biosynthesis (16.98%–17.35%), Fatty Acid and Lipid Biosynthesis (12.24%–16.12%), Nucleoside and Nucleotide Biosynthesis (13.80%–14.17%), Carbohydrate Biosynthesis (6.97%–7.37%), TCA Cycle (6.55%–6.89%), Fermentation (6.59%–6.79%), Respiration (3.91%–4.02%), Nucleoside and Nucleotide Degradation (3.35%–4.09%), and Glycolysis (3.61%–3.73%).

Compared with CK, R1 and R5 exhibited significantly higher relative abundances in several functional pathways, including Cofactor, Prosthetic Group, Electron Carrier, and Vitamin Biosynthesis; Fatty Acid and Lipid Biosynthesis; Nucleoside and Nucleotide Biosynthesis; Carbohydrate Biosynthesis; the TCA Cycle; and Nucleoside and Nucleotide Degradation. Notably, the R5 group showed even higher relative abundances in Cofactor, Prosthetic Group, Electron Carrier, and Vitamin Biosynthesis; Fatty Acid and Lipid Biosynthesis; and the TCA Cycle compared to R1. This finding suggests that the long-term cultivation of *A. donax* cv. Lvzhou No.1 can enhance bacterial metabolic functions in saline-alkali soils.

### 3.7 Effects of soil nutrients on microbial community diversity

**Figure 8:**
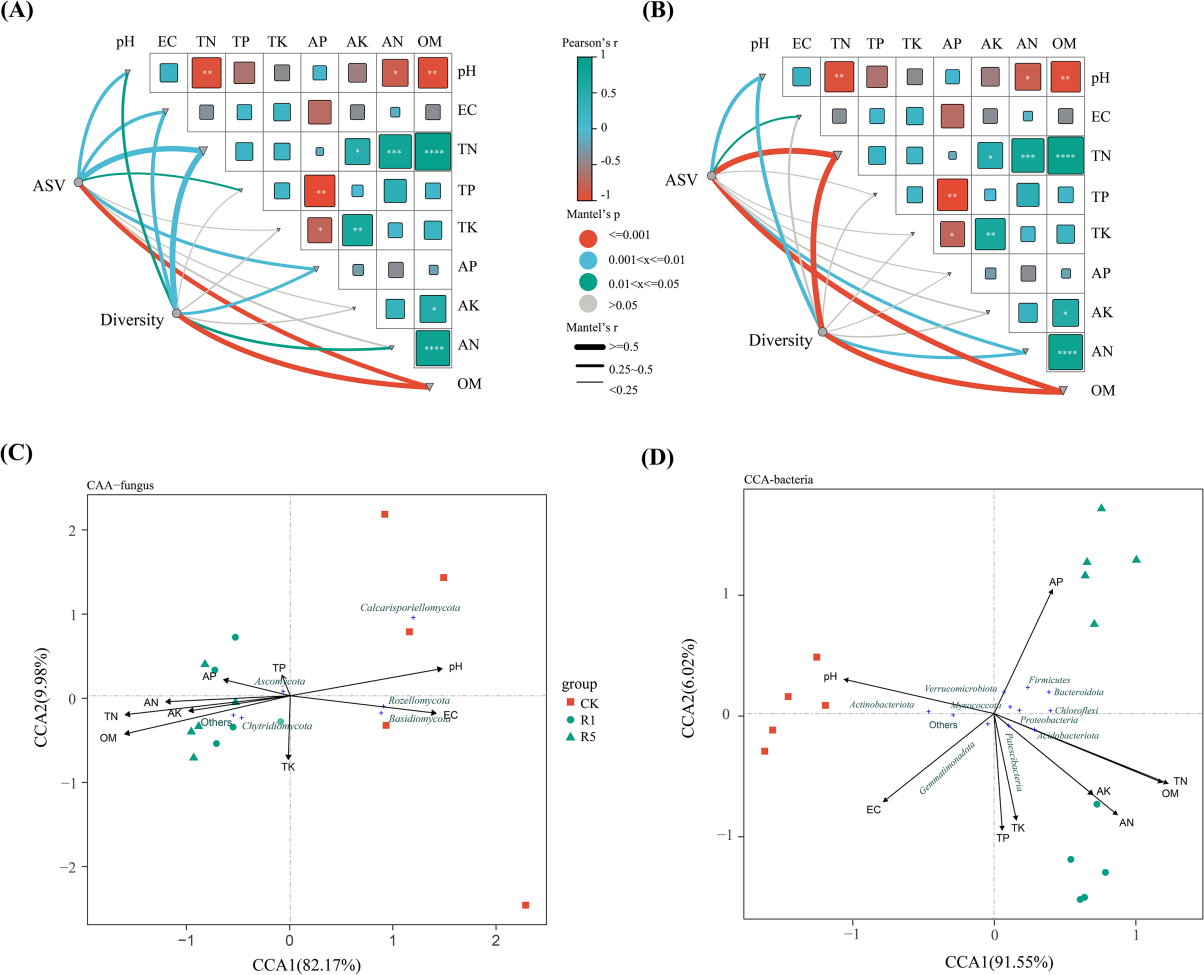
Dependent relationship between soil environmental factors and fungil communities **(A)**, bacterial communities **(B)** on Mantel test analysis; the CCA analysis of fungil **(C)** and bacterial **(D)** microbial community and environmental variables. CK refers to the blank control; R1 indicates one years of cultivation; and R5 denotes five years of cultivation. *, **, *** and **** exhibit significance at the levels of *p* < 0.05, *p* < 0.01, *p* < 0.001 and *p* < 0.0001, respectively.

To identify the key soil chemical properties driving shifts in soil bacterial and fungal community composition, nine chemical properties—AN, AP, AK, OM, TN, TP, TK, EC, and pH—were selected as explanatory variables. Microbial abundance at the phylum level in the rhizosphere soils of CK, one-year (R1), and five-year (R5) *A. donax* cv. Lvzhou No.1 plots served as the response variable. Canonical Correspondence Analysis (CCA) and Mantel tests were employed to examine the relationships between soil chemical properties and microbial communities (**Figure 8**).

Correlation analysis of soil chemical properties (**Figures 8A, B**) revealed strong interrelationships: AN was positively correlated with TN (r = 0.82, *p* < 0.001), OM was positively correlated with TN (r = 0.93, *p* < 0.001) and AN (r = 0.87, *p* < 0.001), and AK was positively correlated with TK (r = 0.74, *p* < 0.01), TN (r = 0.57, 0.01 < *p* < 0.05), and OM (r = 0.61, 0.01 < *p* < 0.05). Soil pH exhibited significant negative correlations with TN (r = -0.71, *p* < 0.01), OM (r = -0.72, *p* < 0.01), and AN (r = -0.61, 0.01 < *p* < 0.05). AP was negatively correlated with TP (r = -0.76, *p* < 0.01) and TK (r = -0.55, 0.01 < *p* < 0.05). The analysis of microbial-soil associations showed that fungal abundance was significantly influenced by TN (r = 0.57, *p* < 0.01), pH (r = 0.32, *p* < 0.01), EC (r = 0.32, 0.01 < *p* < 0.05), AP (r = 0.30, *p* < 0.01), and OM (r = 0.46, *p* < 0.001), whereas fungal diversity was primarily regulated by EC (r = 0.37, *p* < 0.01), AP (r = 0.27, *p* < 0.01), TN (r = 0.63, *p* < 0.01), and OM (r = 0.46, *p* < 0.001). Bacterial abundance was influenced by pH (r = 0.48, *p* < 0.01), TN (r = 0.89, *p* < 0.001), AN (r = 0.41, *p* < 0.01), and OM (r = 0.81, *p* < 0.001), with bacterial diversity similarly regulated by these factors (pH: r = 0.51, *p* < 0.01; TN: r = 0.88, *p* < 0.001; AN: r = 0.38, *p* < 0.01; OM: r = 0.82, *p* < 0.001). These results indicate that TN, OM, AN, and pH are key drivers of rhizosphere microbial composition and diversity under the cultivation of *A. donax* cv. Lvzhou No.1.

The results of Canonical Correspondence Analysis (CCA) (**Figures 8C, D**) further elucidate the relationship between microbial community structure and soil chemical properties. For the bacterial community, axes 1 and 2 explained 91.55% and 6.02% of the total variance, respectively, with a cumulative explanation rate of 97.57%. Several soil nutrient indicators showed significant correlations with bacterial community structure. TN (r² = 0.94, *p* < 0.001), OM (r² = 0.88, *p* < 0.001), AN (r² = 0.73, *p* < 0.01), and AK (r² = 0.46, *p* < 0.01) had high explanatory power for community variation and were all statistically significant. Meanwhile, soil pH (r² = 0.6136, *p* < 0.01) and EC (r² = 0.55, *p* < 0.05) were also significantly correlated with bacterial community distribution (**Supplementary Table S3**). At the taxonomic level, Actinobacteriota and Gemmatimonadota were primarily correlated with pH and EC gradients, while Acidobacteriota and Patescibacteria were more influenced by TN, OM, AK, and AN. Verrucomicrobiota was more closely related to changes in AP.

For the fungal community, axes 1 and 2 explained 82.17% and 9.98% of the variance, respectively, with a cumulative explanation rate of 92.15%. There were significant differences in the explanatory power of various environmental factors on fungal community structure. Specifically, pH (r² = 0.54, *p* = 0.007), EC (r² = 0.49, *p =* 0.019), TN (r² = 0.62, *p* = 0.001), and OM (r² = 0.66, *p* = 0.001) were significantly correlated with fungal community structure, whereas the explanatory power of TP, TK, AP, AK, and AN was lower and did not reach statistical significance (**Supplementary Table S4**). Ascomycota and Chytridiomycota showed significant positive correlations with OM and TN, while Basidiomycota and Rozellomycota were more associated with pH and EC, reflecting the differential response of different fungal taxa to soil physicochemical gradients. Furthermore, in both bacterial and fungal communities, samples from all treatments were primarily distributed in areas aligned with the direction of nutrient vectors, further suggesting that soil nitrogen content, potassium levels, organic matter, and pH are key environmental factors driving changes in the rhizosphere microbial community structure of *A. donax* cv. Lvzhou NO.1 under saline-alkali soil conditions.

## 4 Discussion

### 4.1 Impact of *Arundo donax* cv.Lvzhou No.1 cultivation on soil nutrients in coastal saline-alkaline land

Significant progress has been made in the improvement of extensive saline-alkali soil resources. Traditional physicochemical methods, such as drainage, salt washing, and the application of ameliorants (e.g., gypsum, ferrous sulfate), can rapidly reduce salinity and alkalinity. However, these approaches are often associated with high costs, considerable environmental risks, and the potential for secondary pollution. Therefore, phytoremediation is considered one of the most effective and sustainable methods for improving saline-alkali land. Soil, as a vital ecosystem, not only provides essential materials and energy for terrestrial life systems but also directly influences soil fertility, crop growth, and ecological functions through its physicochemical properties.

To minimize potential confounding effects of fertilization, the same fertilization regime (organic fertilizer applied at establishment and inorganic fertilizer used as topdressing thereafter) was implemented across all treatments, including CK, R1, and R5. Therefore, differences in soil physicochemical properties among CK, R1, and R5 are unlikely to be explained by fertilization alone, but instead reflect the duration-dependent effects of *A. donax* cv. Lvzhou No.1 cultivation on soil nutrient retention and rhizosphere processes. Under this controlled fertilization background, we found that with increasing planting duration of *A. donax* cv. Lvzhou No.1, soil pH significantly decreased, whereas OM, TN, AN, and AK significantly increased. Despite identical fertilizer inputs, soil fertility indices still showed a clear gradient (R5 > R1 > CK for OM, TN, and AN), suggesting that long-term vegetation establishment and associated rhizodeposition and litter inputs enhanced nutrient accumulation beyond the baseline fertilization effect. The potential mechanisms may include increased litter return by perennial vegetation, enhanced recruitment of beneficial microorganisms via root exudates, and improved soil structure and microbial decomposition functions, thereby accelerating organic matter turnover and nutrient recycling and ultimately promoting OM and nutrient accumulation (Ledo et al., 2020; Li et al., 2024; Ruf and Emmerling, 2020). Notably, this study found that the AP content in the rhizosphere soil of five-year-old *A. donax* cv. Lvzhou No.1 was significantly lower than that in one-year-old plots, while its TP content was significantly higher. This phenomenon is not contradictory but reflects the dynamic distribution process between different phosphorus pools in the soil. Previous studies have shown that total phosphorus in soil consists of various forms of phosphorus, with only a small portion existing as available phosphorus that can be directly absorbed by plants. The remaining phosphorus is stored in the soil as organic phosphorus or mineral-bound phosphorus (Gustafsson et al., 2023; Spohn, 2024).

Under the highly alkaline saline-alkali soil conditions studied in this research, with the extension of planting duration, phosphorus in the soil becomes more likely to combine with calcium ions to form insoluble calcium-bound phosphorus (Ca-P). This process results in the conversion of available phosphorus into medium- and low-activity phosphorus pools. Even though total phosphorus continues to accumulate, its bioavailability may still decrease (Hinsinger, 2001). Additionally, five-year-old *A. donax* cv. Lvzhou NO.1 has adapted well to the saline-alkali environment and established a stable growth and heading cycle. Its annual phosphorus absorption demand is significantly higher than that of one-year-old plants, accelerating the depletion of the soil’s readily available phosphorus pool. Therefore, the phenomenon may be the result of long-term vegetation restoration, where litter input and nutrient cycling promote the accumulation of total soil phosphorus, while enhanced phosphorus fixation and continuous plant absorption jointly lead to the relative depletion of the available phosphorus pool.

### 4.2 Effects of *Arundo donax* cv. Lvzhou No.1 on the species composition and diversity characteristics of soil microorganisms in coastal saline-alkaline soil

Plant-microbe interactions shape rhizosphere community assembly through selective recruitment driven by root exudates and rhizodeposition (Pulleman et al., 2012; Wu et al., 2023). In our study, the cultivation of *A. donax* cv. Lvzhou No.1 significantly increased the overall microbial alpha diversity compared with the blank control, and the richness and diversity of bacterial communities were consistently higher than those of fungal communities. Such a pattern is consistent with plant-mediated enrichment of beneficial bacterial taxa and the potential suppression of some pathogenic fungi (Maarastawi et al., 2018; Wang et al., 2018). Moreover, both the number of detected taxa and alpha diversity increased with planting duration, likely reflecting the combined effects of (i) an expanded rhizosphere “hotspot” created by root exudates and mucilage, and (ii) the progressive improvement of soil nutrient and organic matter status with longer cultivation (Buée et al., 2009; Dowarah et al., 2025).

PERMANOVA based on Bray-Curtis dissimilarity showed that *A. donax* cv. Lvzhou No.1 cultivation explained less variation in fungal community composition than in bacterial composition (R² = 0.48 vs. 0.72), indicating a comparatively weaker compositional response of fungi to cultivation over the studied time frame. Meanwhile, fungi exhibited lower alpha diversity than bacteria in our dataset. Taken together, these observations are consistent with previous reports that fungal communities often display lower species diversity than bacterial communities and may respond more conservatively at the community-wide level, potentially reflecting higher structural stability (Chen et al., 2020). At the phylum level, Ascomycota dominated the rhizosphere fungal community and increased with planting duration, consistent with common soil fungal distribution patterns (Wang et al., 2018). In contrast, the relative abundance of Rozellomycota and Basidiomycota changed significantly with planting years, with Rozellomycota decreasing and Basidiomycota showing an increasing trend. These shifts likely reflect the progressive modification of rhizosphere microhabitats and substrate quality associated with longer cultivation. Mantel analysis indicated that fungal abundance and diversity were associated with EC, TN, and OM, while CCA showed that Ascomycota and Calcarisporiellomycota were positively correlated with AP, AN, AK, TN, and OM. This implies that prolonged cultivation improves nutrient conditions that support saprotrophic fungal groups. Basidiomycota, often linked to decomposition of more recalcitrant plant litter, may increase as organic matter inputs accumulate over time (Lienhard et al., 2014).

Compared with fungi, bacterial communities exhibited a stronger community-wide response to cultivation treatments, as indicated by PERMANOVA based on Bray-Curtis dissimilarity (R² = 0.72). At the phylum level, cultivation significantly increased the relative abundance of Proteobacteria, Acidobacteriota, and Chloroflexi, with Proteobacteria showing an increasing trend with planting duration. Mantel analysis suggested that bacterial abundance and diversity were jointly associated with pH, TN, AN, and OM, and CCA further indicated that Acidobacteriota was positively correlated with OM, TN, AN, and AK but negatively correlated with pH and EC. Together, these results support that *A. donax* cv. Lvzhou No.1 cultivation rapidly promotes nutrient accumulation and alleviates the alkaline/saline stress environment, thereby favoring bacterial groups linked to organic matter turnover and nutrient cycling (Mao et al., 2019; Kalam et al., 2020; Goncalves et al., 2024; Górska et al., 2024). The gradual decrease in pH and increase in OM with planting duration may provide additional resources that sustain Acidobacteriota through flexible substrate utilization (Kalam et al., 2020; Goncalves et al., 2024). In addition, Firmicutes increased with planting duration and was positively correlated with AP, suggesting that phosphate-solubilizing members may contribute to enhanced P availability in the rhizosphere. Collectively, these patterns suggest that bacteria respond rapidly to short-term changes in soil nutrients and physicochemical conditions induced by *A. donax* cv. Lvzhou No.1 cultivation, whereas fungal responses may be more constrained by substrate quality and longer-term successional processes.

### 4.3 Effects of *Arundo donax* cv.Lvzhou No.1 cultivation on rhizosphere microbial indicator species in coastal saline-alkali soil

To further pinpoint the taxa driving the community shifts observed in Section 4.2, we applied LEfSe to identify differentially enriched indicator taxa across CK, R1, and R5. The LEfSe results indicated pronounced fungal differentiation following cultivation. In the R1 group, the rhizosphere soil was enriched with Ascomycota-affiliated taxa, particularly the order Hypocreales, which includes many saprotrophic or facultatively saprotrophic fungi with strong cellulose and hemicellulose degradation potential (Schmoll et al., 2009; Berlemont et al., 2014). This enrichment supports that early-stage cultivation stimulates decomposer activity via increased rhizodeposition and litter inputs. In the R5 group, *Fusarium* was significantly enriched. While *Fusarium* is frequently discussed as a pathogen-associated genus (Zhou et al., 2024), its ecological role can be context-dependent, with functional plasticity reported across species/strains and environments (Huang et al., 2015). Considering the increased OM and the overall enrichment of saprotrophic fungi in R5, the enrichment of *Fusarium* here may reflect a shift toward decomposition and nutrient transformation under high organic inputs, although potential pathogenic risks warrant further functional validation (Bernardo-Cravo et al., 2020; Nikitin et al., 2023). The enrichment of Sordariomycetes in R5 further supports a successional transition toward fungal groups involved in lignocellulose decomposition under prolonged cultivation (Miao et al., 2022).

In the bacterial community, the CK rhizosphere soil showed enrichment of Actinobacteriota, consistent with their competitive advantage under relatively oligotrophic conditions (Razgour et al., 2013). In the R1 group, bacterial diversity increased markedly and taxa from Acidobacteriota and Proteobacteria were enriched, consistent with enhanced nutrient inputs from root activity and rhizodeposition (Morrissey et al., 2017; Mao et al., 2019; Matteoli et al., 2022). In contrast, R5 was enriched with *Chloroflexi* taxa, including filamentous groups linked to degradation of complex organic materials, suggesting a later-stage specialization toward decomposition under sustained OM accumulation (Bovio-Winkler et al., 2023). Collectively, the LEfSe results support a successional trajectory from oligotrophic-associated taxa in CK to copiotrophic and diverse bacterial assemblages in R1, and further toward specialized decomposer-dominant communities in R5, with fungal indicators reflecting an increasingly lignocellulose-oriented decomposition niche under prolonged cultivation.

### 4.4 Functional prediction of microbial communities

Saprotrophic fungi are capable of decomposing complex organic matter such as plant residues and animal manure into small organic molecules and inorganic nutrients, making them key functional groups that drive soil organic matter turnover and nutrient release. In contrast, pathogenic fungi primarily extract nutrients from host tissues to sustain their growth and may negatively impact plant growth. The results of this study show that the soil fungal community in the study area is predominantly composed of saprotrophic and pathogenic fungi. compared to the control site, the relative abundance of saprotrophic fungi and undefined saprotrophs significantly increased after the planting of *Arundo donax* cv. Lvzhou No.1, while the relative abundance of plant pathogenic fungi significantly decreased, with the decrease further intensifying as the planting period lengthened. This suggests that the planting of *A. donax* cv. Lvzhou No.1 modifies the rhizosphere environment, promoting the enrichment of organic matter-decomposing functional groups and, to some extent, inhibiting the expansion of potential pathogenic fungi, thereby improving the soil micro-ecosystem (Andargie et al., 2023).

Additionally, Ascomycota and Basidiomycota, as typical saprotrophic fungal groups, play a crucial role in the decomposition of recalcitrant organic compounds, including lignin, cellulose, and keratin. These fungi significantly influence carbon cycling and nutrient redistribution in terrestrial ecosystems (Beimforde et al., 2014; Floudas et al., 2012). In this study, the relative abundance of these fungal groups and their associated saprotrophic functions significantly increased, indicating that the planting of *Arundo donax* cv. Lvzhou No.1 provides a stable carbon source to microorganisms through continuous input of litter and root residues. This, in turn, enhances soil organic matter decomposition and nutrient return processes, which aligns with the observed increase in soil organic matter and major nutrient content with the length of planting years. The bacterial functional prediction based on microbial community sequence data indicates that the metabolic potential of the soil bacterial community in the study area is primarily focused on biosynthesis and energy metabolism-related pathways. Compared to the control site, after the planting of *Arundo donax* cv. Lvzhou No.1, the relative abundance of core metabolic pathways, including cofactor and vitamin synthesis, fatty acid and lipid synthesis, amino acid synthesis, and the tricarboxylic acid (TCA) cycle, significantly increased, with the highest levels observed in the five-year-old sample (**Figure 7**). The TCA cycle serves as a central metabolic pathway for microorganisms to acquire energy and carbon skeletons. Its enhancement typically reflects an increase in microbial ability to utilize organic substrates and overall metabolic activity (He et al., 2024; Floudas et al., 2012). The enhancement of fatty acid and cofactor synthesis pathways contributes to the construction of microbial cell membranes, stabilization of enzyme systems, and increased resistance to stress, thereby enhancing microbial survival and functional expression in saline-alkali stress environments (El-Halmouch, 2019; Wang et al., 2024a).

Therefore, the cultivation of *A. donax* cv. Lvzhou No.1 enhances the influx of organic substrates and improves the rhizosphere environment, thereby promoting the establishment of a microbial functional network focused on energy metabolism and biosynthesis. This process accelerates the decomposition of organic matter, nutrient mineralization, and recycling processes in the soil, thereby driving the sustained improvement of saline-alkali soils at the functional level.

## 5 Conclusion

This study reveals the dynamic changes in soil nutrients and microbial communities under different planting durations of *A. donax* cv. Lvzhou NO.1. The cultivation of *A. donax* cv. Lvzhou No.1 significantly reduced soil pH and salinity, while increasing OM and key nutrients, such as TN, AN and AK. These indicators showed significant improvement with extended planting durations. Soil microbial diversity significantly increased, with Ascomycota being the dominant fungal phylum and Proteobacteria being the predominant bacterial phylum. Diversity continued to rise with prolonged planting duration. Functional prediction analysis showed that the relative abundance of saprotrophic fungi and key metabolic pathways, including fatty acid and cofactor synthesis and the tricarboxylic acid cycle, increased with planting duration, significantly regulating microbial functions. Soil TN, OM, AN, and AK are the primary environmental factors driving changes in microbial community structure. In conclusion, *A. donax* cv. Lvzhou No.1 effectively improved the soil of coastal saline-alkali land by enhancing soil physicochemical properties and microbial community structure. As planting duration increased, the improvement became more pronounced, providing important references for the use of *A. donax* cv. Lvzhou NO.1 in ecological restoration and agricultural production in saline-alkali soils.

Although this study explored the impact of the planting duration of *A. donax* cv. Lvzhou No.1 on soil nutrients and microbial communities. However, factors such as fertilization timing and interannual climate variations may influence the results. The extension of planting duration may be accompanied by the accumulation of fertilization effects, while climate change, including fluctuations in precipitation and temperature, may also impact soil and microbial communities. Therefore, future research should better control fertilization and climate variables to accurately assess the independent effects of planting duration on soil and microbial communities, while integrating multi-omics technologies to further explore the underlying mechanisms.

## Data Availability

The data supporting this study are available from the corresponding author upon reasonable request. Sequencing data have been deposited in the NCBI Sequence Read Archive (SRA) under BioProject accession number PRJNA1396986 (BioSamples: SAMN54407995–SAMN54407997).
