# Supplementary material for: Rhizosphere soil fertility and microbial community characteristics of Arundo donax cv. Lvzhou No.1 in coastal saline-alkali soils
Source: Front Plant Sci. 2026 Feb 24;17:1745488. doi: 10.3389/fpls.2026.1745488 (PMC12971683; doi:10.3389/fpls.2026.1745488)
Supplement: Supplementary file 1 [file Table1.docx]

Supplementary Material

## 1 Supplementary Figures


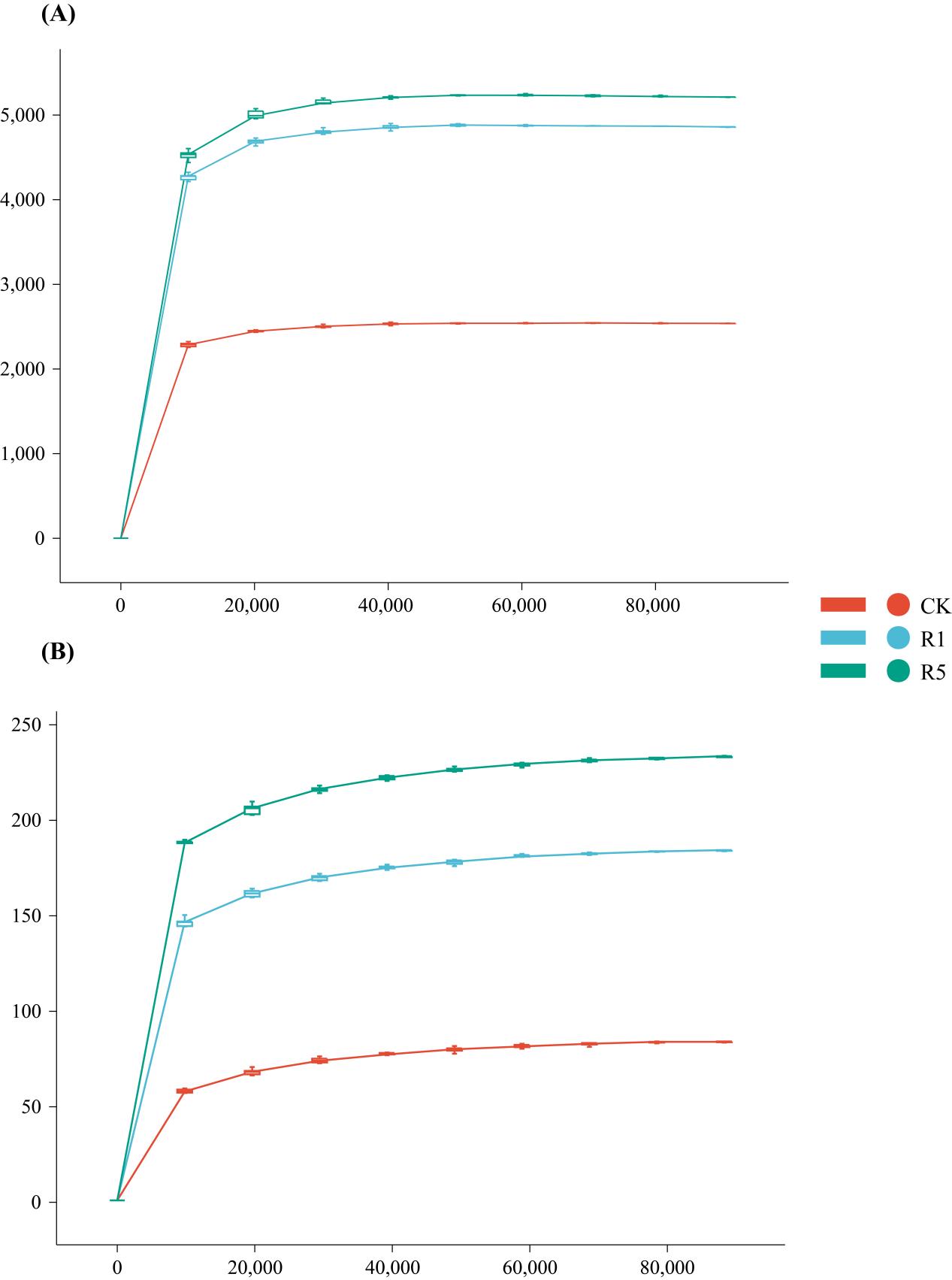


**Supplementary Figure 1.** Dilution curve of soil fungal (A). Dilution curve of soil bacteria (B). Note: CK, the blank control; R1, one-year cultivation; R5, five-year cultivation(Hu et al., 2026; Lei et al., 2025).

**References**

Hu X, Liu C, Xu J, et al., 2026. Seasonal dynamics of microbial diversity and ecological functions in the river estuary area of loushan river[J/OL]. Journal of Hydrology, 664: 134464. DOI:10.1016/j.jhydrol.2025.134464.

Lei Y, Jiang F, Di X, et al., 2025. Cultivating Dictyophora indusiata with Cenchrus fungigraminus and seafood mushroom spent substrate enhanced soil nutrient accumulation and microbial community stabilisation[J/OL]. Environmental Technology & Innovation, 38: 104200. DOI:10.1016/j.eti.2025.104200.
