# Supplementary material for: Rhizosphere soil fertility and microbial community characteristics of Arundo donax cv. Lvzhou No.1 in coastal saline-alkali soils
Source: Front Plant Sci. 2026 Feb 24;17:1745488. doi: 10.3389/fpls.2026.1745488 (PMC12971683; doi:10.3389/fpls.2026.1745488)
Supplement: Supplementary file 2 [file Table2.docx]

| Tab. 1. Chemical Properties of Soil for *Arundo donax* cv.Lv zhou No.1 in Coastal Saline-Alkali Land at Different Planting Years | | | | | | | | | |
| --- | --- | --- | --- | --- | --- | --- | --- | --- | --- |
| Measures | pH | Ec  （mS/cm） | OM （g/kg） | TN （g/kg） | TP （g/kg） | TK (g/kg) | AP (mg/kg) | AK (mg/kg) | AN (mg/kg) |
| CK | 9.214 ± 0.07 a | 86.82 ± 9.24 a | 1.57 ± 0.13 c | 0.10 ± 0.004 c | 0.34 ± 0.01 ab | 2.29 ± 0.20 ab | 5.28 ± 0.48 b | 47.78 ± 4.70 b | 9.23 ± 1.49 c |
| R1 | 9.108 ± 0.05 b | 73.28 ± 2.97 b | 2.50 ± 0.33 b | 0.14 ± 0.01 b | 0.33 ± 0.01 b | 2.14 ± 0.14 b | 6.12 ± 0.42 a | 52.87 ± 0.10 ab | 12.348 ± 2.41 b |
| R5 | 9.074 ± 0.04 b | 83.2 ± 5.93 a | 3.00 ± 0.21 a | 0.17 ± 0.004 a | 0.36 ± 0.02 a | 2.56 ± 0.30 a | 5.07 ± 0.34 b | 66.62 ± 16.73 a | 16.57 ± 2.44 a |

Note: Values are means ± SD (n = 5 replications). CK, the blank control; R1, one-year cultivation; R5, five-year cultivation. Different lowercase letters in the same column indicate significant difference used by Duncan’s new multiple range test (*p*<0.05).

| Tab. S1. Statistical Data of Soil Microbial Sequencing for *Arundo donax*.cv .Lvzhou No.1 in Coastal Saline-AlkaliL and at Different Planting Years | | | | | | |
| --- | --- | --- | --- | --- | --- | --- |
| Funguild | | | | Bacteria | | |
| Measures | Raw number | Clean number | ASVs Effective number | Raw number | Clean number | ASVs Effective number |
| CK | 123428.4 | 101039.4 | 83.98 | 129433 | 112883.4 | 2528.3 |
| R1 | 136170.2 | 108145.6 | 184.26 | 130443.8 | 104335 | 4841.82 |
| R5 | 138048 | 107939.6 | 233.4 | 133864.2 | 106156.2 | 5187.02 |

| Table.S2 PERMANOVA Analysis of Differences Between Treatments | | | | | | | |
| --- | --- | --- | --- | --- | --- | --- | --- |
| Community type | Source | Df | SumsOfSqs | MeanSqs | F.Model | R2 | Pr(>F) |
| Fungi | Treat | 2 | 1.588524 | 0.794262 | 5.470518 | 0.47692 | 0.001 |
|  | Residuals | 12 | 1.742274 | 0.14519 | NaN | 0.52308 | NaN |
|  | Total | 14 | 3.330798 | NaN | NaN | 1 | NaN |
| Bacteria | Treat | 2 | 2.934709 | 1.467355 | 15.145946 | 0.716258 | 0.001 |
|  | Residuals | 12 | 1.162572 | 0.096881 | NaN | 0.283742 | NaN |
|  | Total | 14 | 4.097281 | NaN | NaN | 1 | NaN |

| Table.S3 CCA-Bacteria Results: The Relationship Between Environmental Factors and Species Community Structure | | | | |
| --- | --- | --- | --- | --- |
| Environmental Factors | CCA1 | CCA2 | r^2^ | Pr(>r) |
| pH | -0.9665 | 0.2568 | 0.6136 | 0.005 |
| EC | -0.7605 | -0.6494 | 0.5468 | 0.013 |
| TN | 0.9123 | -0.4096 | 0.9373 | 0.001 |
| TP | 0.0885 | -0.9961 | 0.4375 | 0.03 |
| TK | 0.2123 | -0.9772 | 0.3782 | 0.051 |
| AP | 0.3868 | 0.9221 | 0.5687 | 0.004 |
| AK | 0.7487 | -0.6629 | 0.4649 | 0.002 |
| AN | 0.7485 | -0.6631 | 0.7282 | 0.002 |
| OM | 0.9095 | -0.4157 | 0.8829 | 0.001 |

| Table.S4 CCA-Fungus Results: The Relationship Between Environmental Factors and Species Community Structure | | | | |
| --- | --- | --- | --- | --- |
| Environmental Factors | CCA1 | CCA2 | r^2^ | Pr(>r) |
| pH | 0.9786 | 0.2059 | 0.5398 | 0.007 |
| EC | 0.9881 | -0.1537 | 0.4889 | 0.019 |
| TN | -0.9915 | -0.1302 | 0.6215 | 0.001 |
| TP | -0.3167 | 0.9485 | 0.0158 | 0.909 |
| TK | -0.0156 | -0.9999 | 0.1367 | 0.377 |
| AP | -0.9563 | 0.2925 | 0.109 | 0.522 |
| AK | -0.9852 | -0.1712 | 0.2377 | 0.161 |
| AN | -0.9988 | -0.0493 | 0.3486 | 0.075 |
| OM | -0.9644 | -0.2644 | 0.6604 | 0.001 |
